# Current Advances in the Physiological Roles of the Thioredoxin-Like Family of Selenoproteins

**DOI:** 10.1007/s12011-026-05154-x

**Published:** 2026-05-28

**Authors:** Qingzhou Wang, Wen-Hsing Cheng

**Affiliations:** 1 Department of Biochemistry, Nutrition, and Health Promotion, Mississippi State University, Mississippi State, MS 39762 USA; 2 Department of Nutrition and Food Sciences, Texas Woman’s University, Denton, TX 76209 USA

## Abstract

Selenium (Se) is an essential trace element whose physiological functions in redox regulation, immunity, and metabolism are primarily executed by 25 distinct human selenoproteins. While systemic Se status is maintained through a tightly regulated hepatic distribution hierarchy and receptor-mediated transport to vital organs, recent research has significantly advanced our understanding of the specific roles of individual selenoproteins. This review focuses on the thioredoxin-like family, comprising SELENOT, SELENOV, SELENOW, and SELENOH, which share a conserved Rdx fold but perform diverse, tissue-specific functions. SELENOT has been implicated in the development and function of catecholaminergic circuits and may contribute to the regulation of calcium homeostasis, while SELENOV appears to play a role in modulating energy balance, adiposity, and hepatic responses to stress resistance. Emerging evidence suggests that SELENOW may play roles in muscle maintenance, bone remodeling, and inflammation resolution, with potential involvement in neuroprotection via regulation of tau homeostasis. Finally, the nuclear-localized SELENOH preserves genomic stability and is involved in cell cycle progression. By integrating findings from molecular pathways and physiological data from knockout mouse models, this review highlights the roles of these four proteins in supporting cellular viability and physiological homeostasis under conditions of oxidative and metabolic stress.

## Introduction

Selenium (Se) is an essential trace element whose physiological functions in redox regulation, immunity, and reproduction are primarily mediated by 25 selenocysteine-containing selenoproteins. The relationship between Se status and human health generally follows a U-shaped curve where both deficiency and excess present significant risks. Severe deficiency can lead to Keshan and Kashin-Beck diseases, while even moderate deficiency is linked to impaired immunity, reduced fertility, thyroid dysfunction, cognitive decline, and defective insulin signaling [1, 2]. Intakes exceeding the 55 µg/day RDA increase risks of type 2 diabetes, while doses significantly above the tolerable upper limit (e.g., 10x the RDA) can lead to selenosis, a condition characterized by alopecia and skin disorders [3]. Supplementation is generally unnecessary for individuals on a balanced diet because while the body does not regulate selenium uptake, it effectively detoxifies and excretes excess selenium through breath and urine [4].

Systemically, Se distribution is tightly regulated by hepatic processing and receptor-mediated transport to peripheral tissues. As the central hub, the liver stores Se within glutathione peroxidase 1 (GPX1) and synthesizes selenoprotein P (SELENOP), the primary transport vehicle. During deficiency, the liver reduces its own GPX1 content to prioritize the production and export of SELENOP. This delivery system relies on specific cell-surface receptors to ensure targeted, tissue-specific uptake [5].

To manage Se scarcity, the body utilizes a biological hierarchy that prioritizes vital organs, such as the brain and testes, over non-essential tissues like the liver and kidneys. This tissue-level prioritization appears to act as a primary safeguard for preserving critical biological functions during periods of low Se intake [6]. The delivery of Se in SELENOP to high-priority organs is facilitated by organ-specific expression of the SELENOP receptors apoER2 (also known as LRP8) and megalin [7, 8]. While apoER2 enables Se retention mainly in the brain and testes, megalin aids in reclaiming Se from renal filtrate to minimize systemic loss. Together, these receptors ensure that Se is allocated according to functional importance, protecting the most vital physiological systems.

Upon cellular uptake, selenocysteine is released from SELENOP through lysosomal proteolysis. Subsequently, selenocysteine β-lyase decomposes selenocysteine into alanine and selenide and transfers the latter to selenophosphate synthetase 2 (SEPHS2). SEPHS2 then catalyzes the synthesis of selenophosphate (H_2_SePO_3_^−^), the essential Se donor required for the biosynthesis of selenocysteine-specific tRNA. This pathway is a cornerstone of the canonical process to co-translationally incorporate selenocysteine, ensuring the synthesis of the 25 known human (24 in rodents) selenoproteins [1]. Interestingly, recent research has expanded this model by identifying an alternative pathway that operates independently of selenocysteine β-lyase. In this mechanism, the enzyme peroxiredoxin 6 (PRDX6) has been shown to capture selenide and deliver it to SEPHS2. This PRDX6-mediated pathway has attracted attention because of its specialized role in regulating ferroptosis, an iron-dependent form of programmed cell death driven by lipid peroxidation [9–11]. By supporting the efficient synthesis of glutathione peroxidase 4, the primary enzyme involved in the decomposition of lipid hydroperoxides, this alternative pathway may contribute to cellular defense against oxidative damage. The discovery of the PRDX6- SEPHS2 interaction suggests an additional layer of regulation in Se homeostasis and cellular adaptation to oxidative stress.

At the molecular level, selenoprotein hierarchy is governed by the unique structure of selenoprotein mRNAs, which contain a UGA codon and a selenocysteine insertion sequence (SECIS) in the 3’ untranslated region. The efficiency of selenocysteine insertion is determined by variations in these SECIS elements and their interactions with *trans*-acting factors such as eIF4a3 and nucleolin [1]. During Se deficiency, the body selectively degrades mRNAs of low-priority proteins like GPX1 and SELENOW primarily through nonsense-mediated decay [12]. Meanwhile, high-priority proteins such as GPX4 are maintained because their translation remains efficient even when Se availability is low, ensuring that the most functionally important proteins continue to be synthesized.

## The Thioredoxin-Like Family of Selenoproteins: Insights from Cellular and Clinical Studies

Human selenoproteins are either demonstrated or predicted to possess redox activity. They are characterized into key functional families, including glutathione peroxidases (GPX1-4 and 6), thioredoxin reductases (TXNRD1-3), iodothyronine deiodinases (DIO1-3), and thioredoxin-like family (SELENOT, SELENOV, SELENOW, and SELENOH), alongside 10 others. Dysfunction in these selenoproteins is implicated in a wide array of pathologies [1].

This mini-review focuses on the four members of the thioredoxin-like family, which share a conserved Rdx fold containing a CxxU motif (C, cysteine; x, any amino acid; U, selenocysteine). They exhibit distinct tissue and organelle specificity, and their expression levels vary significantly in response to Se deficiency [13]. Notably, SELENOH and SELENOW occupy a low position in the selenoprotein hierarchy, making them particularly sensitive to Se depletion.

The expression of thioredoxin-like family proteins is further modulated by tissue, sex, and age. For instance, SELENOV expression is largely restricted to the testes and remains negligible in most other tissues. In the brains of male C57BL/6J mice, *Selenow* mRNA levels are approximately 3–7-fold higher than those of *Selenoh* and *Selenot* across multiple regions, including the cerebellum, substantia nigra, cortex, pons, and hippocampus [14]. Age also plays a critical role; in female mice, hepatic *Selenoh* mRNA, but not *Selenow* or *Selenot*, decreases significantly between 18 and 24 months of age. This trend is not observed in the kidney or heart, nor in male mice [15].

### SELENOT

SELENOT appears to play multiple roles in maintaining cellular homeostasis, particularly in the neural, muscular, and cardiac systems. In neuronal cells, it regulates proliferation, apoptosis, and cell-cycle progression. Silencing *SELENOT* in SK-N-SH cells reduces cell proliferation and triggers apoptosis and G1/S arrest. These defects are accompanied by increased FOXO3 and p27 expression and decreased CDK4 expression. Conversely, *SELENOT* overexpression reverses these molecular changes, confirming its role in supporting neuronal growth and survival [16]. Consistent with this, studies in A-172 glioblastoma cells show that SELENOT sustains endoplasmic reticulum Ca²⁺ signaling and restrains the expression of pro-apoptotic proteins; however, it does not alter migration or anchorage-independent growth [17]. These findings highlight the important roles of SELENOT in endoplasmic reticulum homeostasis and its ability to protect cells against stress-induced apoptosis.

Beyond neural cells, SELENOT has been implicated in maintaining metabolic and redox stability in skeletal and cardiac muscle. In myotubes, *SELENOT* overexpression protects against Se deficiency–induced NADPH imbalance, disulfidptosis, and atrophy [18]. This protection is diminished when NADPH regeneration is blocked or mitochondrial ROS are elevated, suggesting that SELENOT may participate in mechanisms that counteract muscle-wasting pathologies. PSELT, a SELENOT-mimetic peptide, restores cytosolic and mitochondrial redox balance in senescent cardiomyocytes through CD36 inhibition [19]. Furthermore, it mitigates palmitate-induced lipotoxicity, cell death, and lipid droplet accumulation in H9c2 cardiomyocytes; however, these protective effects are absent in PSELT analogs lacking selenocysteine [20]. Altogether, these studies suggest that SELENOT functions as a key redox-active selenoprotein involved in maintaining endoplasmic reticulum function, oxidative and Ca²⁺ homeostasis, and proliferation across diverse cell types.

### SELENOV

SELENOV appears to play an emerging role in regulating Se metabolism, cellular stress responses, and disease risk. At the cellular level, primary hepatocytes from SELENOV-deficient mice exhibit increased sensitivity to endoplasmic reticulum stress and oxidative injury, suggesting that SELENOV may contribute to intracellular redox balance and endoplasmic reticulum protection. This vulnerability is evident under diquat and acetaminophen exposure, as SELENOV-knockout hepatocytes display increased cell death (10–40%) and marked reductions in total antioxidant capacity (63–83%), glutathione (26–87%), and superoxide dismutase activity (28–36%). These cells also show more pronounced impairments in cellular respiration and ATP production than wild-type cells. In contrast, SELENOV-overexpressing hepatocytes exhibit improved viability, increased antioxidant capacity, and reduced malondialdehyde and protein carbonyl levels following acetaminophen or H₂O₂ exposure, consistent with a protective role for SELENOV [21].

These cellular findings may help contextualize emerging human genetic associations. The *SELENOV* variant rs4802034 has been linked to increased risks of colorectal adenoma and colorectal cancer in Irish and Czech populations, suggesting that the critical roles of SELENOV roles in redox regulation, endoplasmic reticulum homeostasis, and metabolic signaling may extend to disease susceptibility in humans [22]. Altogether, evidence from nutritional, cellular, and genetic studies supports SELENOV as an important modulator of redox homeostasis.

### SELENOW

In the pathological context of multiple myeloma, a malignancy characterized by osteolytic bone destruction, SELENOW is upregulated in patient-derived peripheral blood mononuclear cells and mature osteoclasts compared with healthy controls. This elevated expression is promoted by the osteoclastogenic factor tumor necrosis factor α (TNFα), which is also increased in these patients. TNFα induces a more marked and sustained activation of extracellular signal-regulated kinase signaling than the physiological stimulator receptor activator of nuclear factor κB ligand, thereby driving excessive SELENOW expression [23]. This suggests that a TNFα and SELENOW feedforward signaling loop may contribute to aberrant osteoclast differentiation and subsequent bone loss.

At the cellular level, SELENOW has been reported to modulate the G1-to-S phase cell cycle transition in epithelial cells. siRNA studies indicate that SELENOW expression peaks during the G1 phase and may facilitate progression into the S phase by downregulating the cyclin-dependent kinase inhibitor p21. This regulation appears to involve the MKK4 pathway, as SELENOW depletion increases total MKK4 and activates downstream effectors, including p38γ, p38δ, and JNK2, which phosphorylate p53 at Ser-33 and promote G1 arrest [24–26].

At the protein level in HEK293 cells, Cys33 of SELENOW has been identified as a target of S-glutathionylation, a post-translational modification enhanced by oxidative stress and catalyzed by glutathione S-transferase π. This modification is crucial because it prevents the formation of a potentially destabilizing intramolecular disulfide bond between Cys33 and Cys87, thereby helping preserve SELENOW function under oxidative conditions. Furthermore, SELENOW participates in a protein-protein interaction network by binding to the adapter protein 14-3-3β, with the interaction mediated through its selenocysteine residue, where it competes with thioredoxin 1 (Trx1) for binding. This competitive interaction suggests a compensatory mechanism through which SELENOW protects Trx1-deficient cells from etoposide-induced cell death. By maintaining signaling through its interaction with 14-3-3β [27, 28], SELENOW reduces cell death markers and restores phosphorylation of AKT (also known as protein kinase B).

### SELENOH

SELENOH was initially identified in fruit flies as the gene *BthD* and later characterized in mice and humans. According to Ensembl and NCBI data, the human *SELENOH* gene is located on chromosome 11 and spans 2,305 bp with four exons and seven splice variants; it encodes a 122-amino acid protein with a selenocysteine at residue 44. In mice, *Selenoh* is located on chromosome 2, spans 1,556 bp with four exons and six splice variants, and encodes a 116-amino acid protein with a selenocysteine at residue 38, sharing ~ 81% sequence identity with its human counterpart. Recombinant SELENOH with a selenocysteine-to-cysteine substitution retains glutathione peroxidase-like activity toward H₂O₂, suggesting that catalytic activity may be partially preserved even without selenocysteine, although the precise contribution of the native selenocysteine residue remains to be fully clarified [29]. SELENOH also contains structural motifs that support biochemical interactions with DNA and nuclear components, including an AT-hook domain, a nuclear localization RKRK motif, and a CxxU motif [30]. These features suggest potential roles in both redox regulation and nucleic acid binding.

Analysis of the human chromosome 11 sequence identified a truncated human splice variant of SELENOH, named H0YE28, which lacks the *N*-terminal 28 amino acids and contains a selenocysteine-to-cysteine substitution [31, 32]. According to NCBI (GenBank: CCP78572.1), another shortened form lacking the first 45 amino acids, including the CXXU motif, exists in American minks. These truncated SELENOH forms are therefore considered non-selenoproteins, as they do not carry a decoded selenocysteine. Experimentally, transfection of a FLAG-tagged *SELENOH* vector in human 293T cells resulted in the expression of both long and short isoform; the latter was notably unresponsive to changes in Se levels in the culture medium [33]. However, it remains unclear whether the short form corresponds to H0YE28, and the mechanism by which cysteine replaces selenocysteine during translation has not yet been established.

As a low-hierarchy selenoprotein, SELENOH is particularly sensitive to Se deficiency. While mRNA expression in adult mice is relatively low, it is more prominent in the brain, thymus, testes, uterus, lung, intestine, and spleen. However, Western blot analysis indicates that protein levels are high in the spleen, moderate in the brain, and low or undetectable in other organs, suggesting organ-specific post-transcriptional and translational regulation [29].

In addition to Se deprivation, *SELENOH* mRNA expression declines during cellular senescence in human WI-38 primary lung fibroblasts [34]. However, it is increased in certain cancer cells (e.g., LLC1, LNCaP, and MSC1 cells) and during mouse embryogenesis (E4.5-E14.5) [29]. Functionally, SELENOH has been implicated in genome stability, redox homeostasis, and resistance to senescence [35]. It localizes to the nucleolus, where it may modulate transcriptional programs associated with oxidative stress. Overexpression studies suggest that SELENOH can induce genes involved in glutathione biosynthesis and phase II detoxification, bind stress-responsive DNA elements, and delay replicative senescence via ATM- and ROS-dependent mechanisms [29, 36]. SELENOH has also been reported to interact with p53, potentially mitigating oxidative DNA damage and attenuating ATM- and p53-mediated DNA damage responses in human HeLa cells [35]. In oxidative-stress models, *SELENOH* overexpression in HT22 mouse hippocampal neurons has bene shown to protect against UVB-induced mitochondrial dysfunction and oxidative stress [37–39]. Its transcription is upregulated by H₂O₂ in HeLa cells [35] and by metal-responsive elements in WISH cells [40]. SELENOH may also influence cell cycle regulation, as its overexpression has been associated with changes in p21 and cyclin E1 (CCNE1) levels, suggesting a role in regulating the G1/S transition and limiting uncontrolled proliferation [41].

**Fig. 1:**
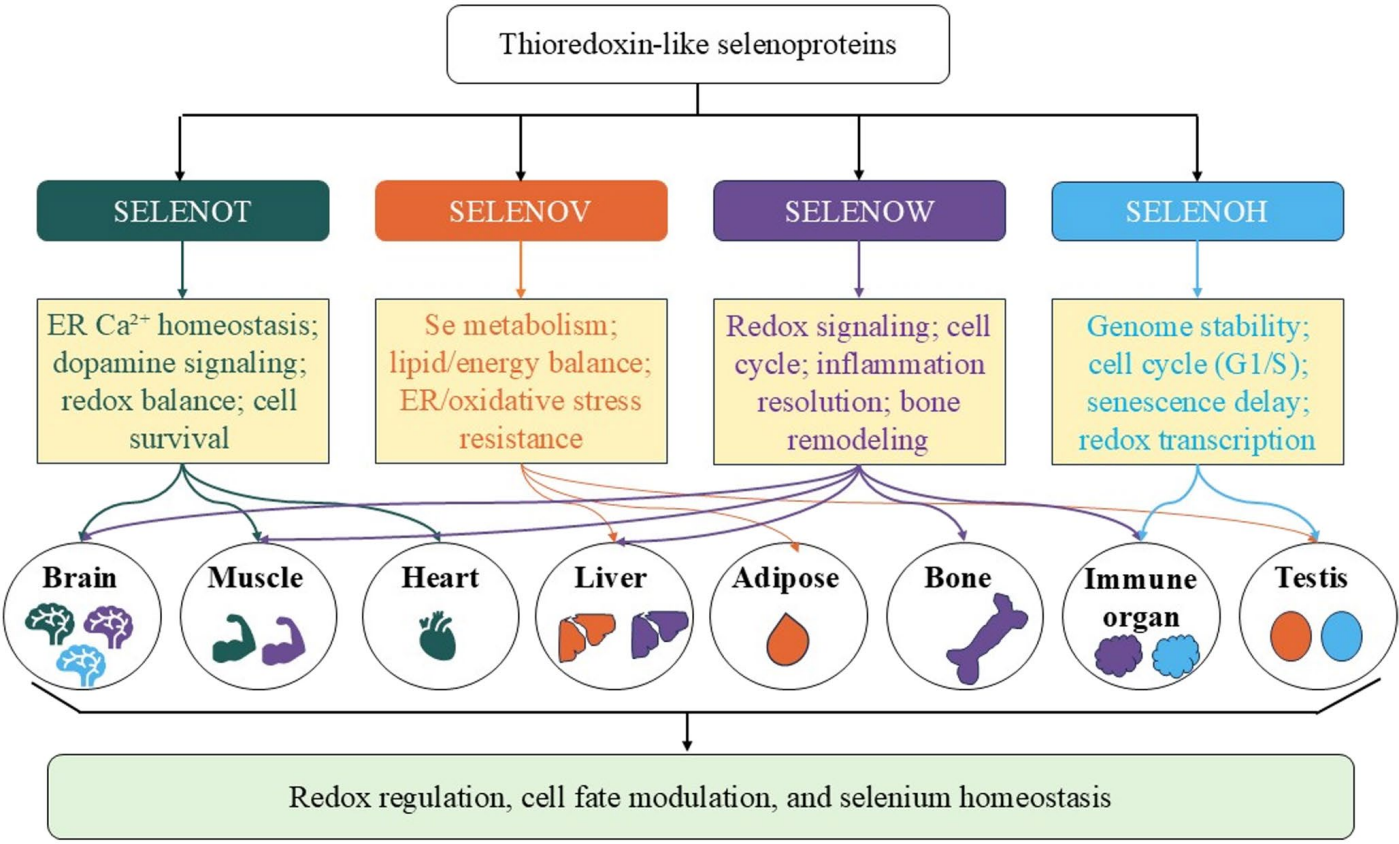
Functional evidence and tissue distribution of thioredoxin-like selenoproteins. This figure illustrates the distribution and biological roles of thioredoxin-like selenoproteins across various tissues. Some of the physiological functions shown in specific organs are inferred from cell studies and await further verification in animal models

The diverse tissue distribution and integrated biological roles of these four family members are summarized in Fig. 1.

## Physiological Roles from Mouse Models

The physiological understanding of the thioredoxin-like family is incomplete and varies widely across members, largely due to differences in the availability of genetic models. While SELENOT and SELENOW have been extensively characterized using whole-body and tissue-specific knockout mouse models, research into SELENOV is more recent, and a SELENOH knockout mouse model has yet to be reported. Among global knockout mouse models, only SELENOT knockout mice exhibit embryonic lethality [42], whereas SELENOV [21, 43] and SELENOW [44] knockout mice are viable. Despite these differences in the depth of available experimental evidence, emerging findings suggest that these proteins share important functions in protecting against oxidative and endoplasmic reticulum stress. These roles are reflected across diverse biological contexts, including the neural and muscular specialization of SELENOT and SELENOW, as well as the metabolic and reproductive Se-buffering functions established for SELENOV in mouse models. Nuclear-targeted roles in genomic stability have also been identified for SELENOH in cellular models, although these findings remain to be validated in animal models.

### SELENOT

Mouse studies indicate that SELENOT is widely expressed throughout the brain, with marked regional differences that suggest site-specific physiological roles. In a mouse model of Parkinson’s disease, *Selenot* mRNA expression is increased in the cerebellum, cortex, and pons, but decreased in substantia nigra [14]. High-resolution 3D mapping combined with tyrosine hydroxylase labeling further suggests that loss of SELENOT compromises catecholaminergic neuron integrity, producing region-specific deficits across dopaminergic and noradrenergic nuclei. These include the A1, A2, A11, and ZI-A13 regions in females, as well as the A12–A14–A15 hypothalamic clusters in both sexes. Such disruptions appear to alter the structural organization of neurotransmitter circuits involved in autonomic regulation, neuroendocrine signaling, and motor control, supporting a role for SELENOT in maintaining catecholaminergic neuronal architecture [45].

Beyond its structural roles, SELENOT also functions as an important regulator of dopaminergic physiology. Mice lacking SELENOT, either globally in the brain or specifically in dopaminergic neurons, display attention-deficit/hyperactivity disorder-like behavioral phenotypes, including hyperlocomotion, impaired recognition memory, repetitive behaviors, and impulsivity. These behaviors are accompanied by neurochemical alterations such as elevated extrasynaptic dopamine, increased dopamine turnover, enhanced striatal excitatory currents, and increased electroencephalography theta power (a method to record electrical activity of the brain). Mechanistically, SELENOT regulates dopaminergic homeostasis through interaction with sarco/endoplasmic reticulum Ca²⁺-ATPase 2, an endoplasmic reticulum calcium pump that controls endoplasmic reticulum–cytosol Ca²⁺ flux. This signaling pathway helps maintain the transcription of the dopamine transporter via nuclear receptor related 1 protein; consequently, dopamine transporter levels are markedly reduced in SELENOT-deficient mice, destabilizing dopamine signaling. Notably, the hyperactivity phenotype can be reversed by amphetamine or methylphenidate treatment, further supporting a central role for SELENOT in maintaining physiological dopamine dynamics [46].

The SELENOT-derived peptide PSELT, which retains the redox-active motif of this protein, reproduces several protective functions of endogenous SELENOT across neural and cardiovascular systems. In peripheral nerve injury models, PSELT accelerates facial nerve regeneration, enhances remyelination, and improves structural integrity, suggesting that the redox activity of SELENOT contributes directly to Schwann cell function and axonal repair [47]. Furthermore, PSELT exerts neuroprotective effects in models relevant to Parkinson’s disease–associated oxidative stress by reducing mitochondrial dysfunction and ROS accumulation while preserving dopaminergic neurons, highlighting its potential as a therapeutic strategy targeting redox imbalance [48].

In the cardiovascular system, SELENOT appears to function as a sensor of cardiomyocyte hypertrophic stress. PSELT treatment mitigates pathological remodeling by reducing cell enlargement and stress-response signaling. These findings suggest that SELENOT-derived peptides may have potential as targeted therapeutic tools for myocardial hypertrophy and heart failure through restoration of redox homeostasis [49]. Collectively, studies of PSELT demonstrate that mimicking the SELENOT redox domain can reproduce many of the protein’s cytoprotective functions, suggesting its translational potential in neurological and cardiovascular diseases.

### SELENOV

Despite being one of the least conserved selenoproteins, mouse studies suggest that SELENOV has important physiological functions spanning energy metabolism, adiposity regulation, and cellular stress resistance. Nutritional studies in *Selenov* knockout mice demonstrate that SELENOV deficiency alters tissue Se homeostasis and affects the expression and activity of several other selenoproteins. Specifically, SELENOV knockout markedly reduces the mRNA levels of *Gpx1*, *Txnrd1*, and *Selenop* in the testis and lowers Se concentrations, while also decreasing Se concentrations and GPX activity in the liver. The knockout additionally induces tissue-specific changes in GPX1 protein abundance, which is elevated in the liver but reduced in the testis. Furthermore, interactions between *Selenov* genotype and dietary fat influence Se distribution and TXNRD activity across multiple tissues. Collectively, these findings support a regulatory role for SELENOV in whole-body Se metabolism [43].

SELENOV knockout mice develop significantly increased body weight (16–19%), driven primarily by a 54% increase in fat mass. This adiposity is associated with coordinated changes in lipid metabolism: lipogenic genes and proteins (*Acc*, *Fas*, *Dgat*, *Lpl*) are upregulated, whereas lipolytic pathways (*Atgl*, *Hs*l, *Ces1d*, *Cpt1a*) are suppressed by 36–89%. These mice also exhibit reduced metabolic activity, including lower oxygen consumption, CO₂ production, and overall energy expenditure (14–23% decreases). Mechanistically, SELENOV interacts with O-GlcNAc transferase (OGT), and its loss reduces OGT protein levels, the enzymatic activity, and O-GlcNAcylation in adipose tissue. A proposed SELENOV–OGT–AMPK regulatory axis may connect these molecular changes to increased fat accumulation and reduced metabolic rate. Collectively, these findings support a role for SELENOV in limiting fat deposition and regulating O-GlcNAc–dependent signaling [50].

SELENOV also appears to contribute to protection against oxidative and endoplasmic reticulum stress. When challenged with the hepatotoxic agents diquat or acetaminophen, knockout mice develop more severe liver injury than wild-type controls. This increased vulnerability is associated with higher serum alanine aminotransferase (ATL) activity and elevated hepatic oxidative damage markers, including malondialdehyde, protein carbonyls, and 3-nitrotyrosine. In addition, knockout mice show increased levels of endoplasmic reticulum stress proteins, particularly binding immunoglobulin protein (BIP/GRP78) and CCAAT-enhancer-binding protein homologous protein (CHOP), together with enhanced activation of apoptosis-related pathways such as focal adhesion kinase (FAK) and caspase-9. Combined with a reduced total antioxidant capacity, the increased hepatic necrosis observed in knockout mice supports a protective role for SELENOV against chemically induced hepatic oxidative and endoplasmic reticulum stress [21].

In summary, SELENOV appears to function in the regulation of whole-body energy balance, adipose signaling, and hepatic stress responses. Its involvement in lipid metabolism and antioxidant defense suggests potential relevance to metabolic disorders and acute liver injury.

### SELENOW

Based on studies using various mouse models, SELENOW appears to be a multifunctional protein involved in redox homeostasis, muscle maintenance, energy metabolism, and neurological function. In the context of sarcopenia and aging, whole-body *Selenow* knockout mice exhibit accelerated muscle mass loss in both age-related and chemically induced atrophy models [44]. This effect has been linked to the loss of SELENOW-mediated suppression of the Ras-related C3 botulinum toxin substrate 1 (RAC1)- mechanistic target of rapamycin (mTOR) cascade. Specifically, through interaction with RAC1, SELENOW helps maintain the balance between protein synthesis and degradation. Conversely, overexpression of SELENOW has been shown to alleviate muscle atrophy, supporting a role for this protein in maintaining muscle mass and regulating aging-associated gene expression.

The role of SELENOW in non-alcoholic fatty liver disease (NAFLD) appears complex, as its loss has been reported to alleviate hepatic steatosis induced by a high-fat diet [51]. Mechanistically, hepatic SELENOW interacts with pyruvate kinase M2 (PKM2) and modulates its nuclear translocation, which in turn promotes the transactivation of hypoxia-inducible factor 1-α (HIF-1α). This cascade has been linked to mitochondrial damage, excessive ROS production, and iactivation of the NLR leucine-rich repeat pyridine domain-containing protein 3 (NLRP3) inflammasome and subsequent pyroptosis. The resulting leakage of mitochondrial DNA may activate the cyclic GMP-AMP synthase (cGAS)-stimulator of interferon genes (STING) signaling pathway in macrophages, potentially exacerbating NAFLD progression. Altogether, in the liver, SELENOW appears to promote hepatocyte apoptosis and pyroptosis through metabolic reprogramming, suggesting a context-dependent role that could be detrimental in NAFLD.

In contrast, studies in Alzheimer’s disease models indicate a potentially protective role for SELENOW in regulating tau homeostasis [52]. SELENOW promotes tau degradation via the ubiquitin–proteasome system by competing with the chaperone protein Hsp70 for tau binding. This interaction facilitates tau ubiquitination and reduces acetylation at Lys281. In *Selenow* knockout mice, the absence of SELENOW is associated with synaptic defects, tau dysregulation, impaired long-term potentiation, and memory deficits. Conversely, SELENOW overexpression in triple-transgenic Alzheimer’s disease mice ameliorates memory impairment and reduces key tau-related pathologies, including decreased levels of 4-repeat tau isoforms, reduced phosphorylation at Ser396 and Ser404, and a lower burden of neurofibrillary tangles. These findings collectively suggest that SELENOW contributes to tau homeostasis and synaptic function, and may represent a potential therapeutic target in Alzheimer’s disease.

SELENOW also plays a significant role in bone remodeling through its regulation of osteoclasts, the cells responsible for bone resorption. Under physiological conditions, osteoclast differentiation is driven by the receptor activator of nuclear factor κB ligand (RANKL)/RANK/p38 mitogen-activated protein kinase (p38 MAPK) signaling pathway, which leads to the downregulation of SELENOW [53]. This repression appears to act as a regulatory checkpoint to limit excessive bone resorption. Consistently, SELENOW knockout suppresses osteoclast formation and results in a high bone mass phenotype. Conversely, SELENOW overexpression enhances osteoclastogenesis by promoting nuclear translocation of NF-κB and nuclear factor of activated T-cells, cytoplasmic 1 (NFATc1) via interaction with 14-3-3γ. This increased activity leads to osteoporosis, indicating that tight regulation of SELENOW expression is important for maintaining physiological bone remodeling and preventing pathological bone loss.

In the context of intestinal inflammation, SELENOW expression appears to support efficient resolution and tissue repair [54]. *Selenow* knockout mice treated with dextran sodium sulfate develop more severe acute colitis, characterized by greater weight loss, shorter colons, and impaired epithelial barrier integrity. This is accompanied by reduced expression of the tight junction protein zonula occludens 1 (ZO-1), increased colonic tumor necrosis factor-α (TNFα), and higher numbers of TNFα-positive macrophages in the lamina propria. Moreover, loss of SELENOW is associated with decreased expression of epithelial markers yes-associated protein 1 (YAP1) and epidermal growth factor receptor (EGFR).

Studies using *Selenow* knockout bone marrow-derived macrophages (BMDMs) further suggest that SELENOW is important for maintaining macrophage redox tone and bioenergetic flexibility [55]. *Selenow*-deficient BMDMs display increased ROS levels and disrupted redox homeostasis. Functionally, these cells show impaired inflammatory resolution, including reduced expression of arginase-1 (a marker of the anti-inflammatory M2 phenotype) and decreased efferocytosis (the clearance of dead neutrophils). Metabolomic analysis indicates that these defects are linked to impaired arginine metabolism. In addition, while wild-type macrophages typically shift toward glycolysis during inflammatory activation, *Selenow* knockout BMDMs show reduced metabolic flexibility and rely more on oxidative phosphorylation even in glucose-rich conditions, suggesting that SELENOW may be required for appropriate metabolic reprogramming during immune responses.

Studies in the brain using *Selenow* knockout mice reveal sex-specific morphological and functional changes. Histomorphological changes have been observed in the amygdala and hippocampus of female *Selenow* knockout mice, accompanied by reduced anxiety-like behavior and impaired contextual fear memory. Comparative proteomic analysis on female knockout mice without fear conditioning show that their hippocampal protein expression partially overlaps with wild-type mice that undergo fear conditioning [56]. These shared differentially expressed proteins are enriched in processes related to ensheathment and oligodendrocyte differentiation. This suggests that SELENOW deletion may affect oligodendrogenesis and myelin maintenance, which are important for the neural circuit plasticity involved in fear memory formation.

## Conclusion and Future Perspectives

Research on SELENOT identifies it as an important contributor to the organization of catecholaminergic circuits and a regulator of calcium-dependent neurotransmission, supporting the structural and functional integrity of dopaminergic and noradrenergic neurons in regions such as the substantia nigra and hypothalamus. A proposed mechanism involves a SELENOT–SERCA2–NURR1 axis, in which SELENOT interacts with the SERCA2 pump to modulate calcium flux, a process required for NURR1-mediated transcription of the dopamine transporter. Accordingly, SELENOT deficiency has been associated with reduced dopamine transporter levels and resulting ADHD-like behavioral phenotypes in mouse models. The redox-active domain, represented by the synthetic peptide PSELT, has been reported to exert cytoprotective effects in several experimental systems, including enhanced peripheral nerve regeneration, attenuation of cardiomyocyte hypertrophy, and protection of neurons in models relevant to Parkinson’s-related oxidative stress. Future studies may help clarify the translational potential of PSELT-based approaches for conditions such as ADHD and Parkinson’s disease, including whether modulation of SELENOT-related redox pathways can influence dopamine homeostasis in humans. It may also be useful to examine how sex hormones influence the observed sex-dependent neuronal deficits. Furthermore, mapping the tissue-specific interactome of SELENOT could help clarify how its functions are coordinated between calcium regulation and redox activity, alongside accessing whether SELENOT expression profiles might have value as exploratory biomarkers for early neurodegenerative or cardiac remodeling processes.

Research into SELENOV characterizes it as a metabolic and homeostatic regulator involved in systemic selenium distribution, adipose tissue expansion, and hepatic responses to acute stress. Mechanistically, SELENOV has been proposed to act as a node in energy balance through a SELENOV–OGT–AMPK axis. Beyond lipid metabolism, it may function as a buffer within the selenoproteome and appears to contribute to the appropriate expression of other key selenoproteins such as GPX1 and TXNRD1, particularly under conditions of nutritional or oxidative stress. In addition, SELENOV has been implicated in protection against oxidative insults, including attenuation of endoplasmic reticulum stress and reduced activation of apoptotic pathway such as FAK/Caspase-9 during hepatic injury. Future research could explore whether the SELENOV–OGT–AMPK axis is amenable to pharmacological modulation in metabolic disease, including whether SELENOV mimetics might help restore O-GlcNAcylation and metabolic function in insulin-resistant states. Given the tissue-specific changes observed within the selenoprotein hierarchy, further work is also needed to clarify how SELENOV influences the mRNA stability or translation of other selenoproteins such as GPX1 and SELENOP. Furthermore, since SELENOV deficiency appears to increase susceptibility to ER stress-induced apoptosis, identifying the specific unfolded protein response pathways that interact with SELENOV may help clarify its role in limiting hepatic injury. Translating these mouse-model findings to human health will require investigating whether SELENOV genetic variants are associated with differences in adiposity or susceptibility to drug-induced liver injury, which may help determine its potential as a biomarker of metabolic and oxidative resilience.

A growing body of recent physiological evidence suggests that SELENOW may function not only as an antioxidant, but also as a regulator tissue-specific metabolic and signaling states. Across diverse physiological systems, SELENOW appears to act through several functional modes, including protective signaling and maintenance, metabolic reprogramming and bioenergetics, and tissue remodeling and repair. Future research should move beyond identifying individual SELENOW targets toward understanding its context-dependent interactions. A key area of interest is the development of tissue-specific SELENOW inhibitors or mimics. Because SELENOW appears to be protective in the brain and muscle but may have detrimental effects in the liver and bone under certain conditions, systemic Se supplementation may involve context-dependent trade-offs. Further work is needed to clarify how SELENOW may function as a regulatory switch between roles such as redox buffering, metabolic regulation, and scaffold-like interactions with proteins including 14-3-3γ and Hsp70. Given reported sex-specific effects in brain morphology and memory, it will also be important to investigate how hormonal environments influence SELENOW expression and whether a selenoprotein hierarchy exists in which SELENOW deficiency triggers compensatory changes in other selenoproteins during metabolic or inflammatory stress. Finally, these mechanistic insights should be translated into clinical studies assessing whether circulating SELENOW levels or specific genetic polymorphisms may serve as potential biomarkers for the progression of sarcopenia, NAFLD, or early-stage Alzheimer’s disease.

Finally, current understanding of SELENOH is based largely on expression studies and functional cellular models. The decline of *SELENOH* mRNA during replicative senescence in WI-38 primary lung fibroblasts suggests a linkage between SELENOH and protection against a persistent DNA damage response [34]. More direct evidence comes from functional studies in *SELENOH* knockdown MRC-5 human primary fibroblasts [35], where SELENOH deficiency leads to accelerated replicative senescence, growth inhibition, and sustained activation of DNA damage signaling, including induction of phospho-ATM at Ser-1981. These findings in primary cell systems are complemented by studies in immortalized and cancer-derived cell lines, including HeLa and HT22 cells, which has further support roles for SELENOH in redox-sensitive genomic stability and cell cycle regulation. However, translating these cellular observations into physiological evidence will require the development of SELENOH knockout mouse models. Such models would help determine whether the genomic stability and anti-senescence functions identified in vitro also contribute to systemic protection in vivo. Future studies should also examine isoform-specific expression, including whether truncated variants such as H0YE28 are endogenously expressed and whether they respond differently to selenium status compared with the full-length protein [33]. In addition, given its distinctive nuclear localization and AT-hook domain, identifying the nuclear targets of SELENOH, including stress-responsive DNA elements and nucleolar interaction partners, will be important for understanding how SELENOH coordinates transcriptional programs involved in oxidative stress resistance and replicative senescence.

## Data Availability

No datasets were generated or analysed during the current study.
